# MMPs and Soluble ICAM-5 Increase Neuronal Excitability within *In Vitro* Networks of Hippocampal Neurons

**DOI:** 10.1371/journal.pone.0042631

**Published:** 2012-08-13

**Authors:** Mark Niedringhaus, Xin Chen, Rhonda Dzakpasu, Katherine Conant

**Affiliations:** 1 Interdisciplinary Program in Neuroscience, Georgetown University Medical Center, Washington, District of Columbia, United States of America; 2 Department of Physics, Georgetown University, Washington, District of Columbia, United States of America; 3 Department of Pharmacology and Physiology, Georgetown University Medical Center, Washington, District of Columbia, United States of America; 4 Department of Neuroscience, Georgetown University Medical Center, Washington, District of Columbia, United States of America; SUNY Downstate Medical Center, United States of America

## Abstract

Matrix metalloproteinases (MMPs) are zinc-dependent endopeptidases that are released from neurons in an activity dependent manner. Published studies suggest their activity is important to varied forms of learning and memory. At least one MMP can stimulate an increase in the size of dendritic spines, structures which represent the post synaptic component for a large number of glutamatergic synapses. This change may be associated with increased synaptic glutamate receptor incorporation, and an increased amplitude and/or frequency of α-amino-3-hydroxyl-5-methyl-4-isoxazole-propionate (AMPA) mini excitatory post-synaptic currents (EPSCs). An associated increase in the probability of action potential occurrence would be expected. While the mechanism(s) by which MMPs may influence synaptic structure and function are not completely understood, MMP dependent shedding of specific cell adhesion molecules (CAMs) could play an important role. CAMs are ideally positioned to be cleaved by synaptically released MMPs, and shed N terminal domains could potentially interact with previously unengaged integrins to stimulate dendritic actin polymerization with spine expansion. In the present study, we have used multielectrode arrays (MEAs) to investigate MMP and soluble CAM dependent changes in neuronal activity recorded from hippocampal cultures. We have focused on intercellular adhesion molecule-5 (ICAM-5) in particular, as this CAM is expressed on glutamatergic dendrites and shed in an MMP dependent manner. We show that chemical long-term potentiation (cLTP) evoked changes in recorded activity, and the dynamics of action potential bursts in particular, are altered by MMP inhibition. A blocking antibody to β_1_ integrins has a similar effect. We also show that the ectodomain of ICAM-5 can stimulate β_1_ integrin dependent increases in spike counts and burst number. These results support a growing body of literature suggesting that MMPs have important effects on neuronal excitability. They also support the possibility that MMP dependent shedding of specific synaptic CAMs can contribute to these effects.

## Introduction

Matrix metalloproteinases (MMPs) are a family of zinc dependent endoproteases that play a role in dynamic processes including cell migration and wound healing [1]. While studies of MMPs in the central nervous system (CNS) have generally focused on injury [2], [3], accumulating evidence supports an important role for these enzymes in normal CNS physiology [4], [5].

Neuronal activity stimulates increased MMP release [6], [7], [8] and we have observed rapid MMP dependent shedding of a neuronal substrate following treatment of cultures with N-methyl-D-aspartic acid (NMDA) [8]. Studies suggest that MMPs exist in perisynaptic vesicles [9], and that MMP release may be soluble NSF attachment protein receptor (SNARE) dependent [10]. This suggests that MMP release will occur with select stimuli that increase intracellular calcium.

In several recent studies, MMP activity has been shown to play a role in learning and memory [11], [12], [13], [14], [15]. While effects are likely influenced by factors including MMP dose and duration, and the developmental stage of neurons, these enzymes have the potential to increase glutamatergic transmission, long-term potentiation (LTP), and measures of hippocampal dependent memory [16]. For example, MMP-9 deficient mice show defects in LTP [11] and antisense oligonucleotides for MMPs can prevent acquisition in the Morris water maze test [13]. In addition, mice that over-express MMP-9 have been shown to display enhanced performance in a spatial task [14].

The mechanisms by which MMPs may contribute to changes that underlie learning and memory are likely multiple and not completely understood. Remodeling of the extracellular matrix has been posited, as has an MMP dependent increase in a matrix fragment that can stimulate integrin dependent phosphorylation of glutamate receptor subunits [11]. Consistent with a role for integrins are studies in which integrin antagonists have blocked MMP dependent changes in dendritic spine shape or LTP [11], [17].

An additional mechanism by which MMPs might rapidly modulate synaptic structure and function would be through their ability to affect an increase in the size of dendritic spines, the post synaptic components for a majority of glutamateric synapses. Indeed, at least one MMP has been shown to stimulate increases in the size of dendritic spines [12]. There is a strong correlation between size of the spine head and strength of the synapse, presumably in part because a larger spine head allows for insertion of more glutamate receptors [18].

In terms of the mechanism(s) by which MMPs could affect an increase in spine size, it is important to consider their potential to cleave specific synaptic adhesion molecules. Of particular interest to spine morphology, is MMP dependent shedding of ICAM-5, an adhesion molecule that is expressed on glutamatergic neurons of the telencephalon. A correlation between developmental shedding of ICAM-5 and spine maturation has been demonstrated [19], and long term NMDA treatment (16 h) of neurons has been associated with both spine enlargement and MMP dependent shedding of this molecule [20]. ICAM-5 is well positioned to be targeted by synaptically released MMPs, and MMP dependent shedding of this CAM is observable within 5 minutes of NMDA application [8]. ICAM-5 shedding could disrupt N and C terminal interactions of the full length molecule that are important to filopodial maintenance [21], and shedding may thus be permissive for spine expansion. A non-mutually exclusive possibility is that the shed N terminal domain could interact with unengaged post synaptic integrins to stimulate actin polymerization within dendrites and thus spine expansion. Integrin signaling plays a role in developmental changes in spine morphology [22], [23], and varied forms of learning related plasticity are thought to be integrin dependent [11], [13], [24]. Consistent with this, we have previously shown that soluble ICAM-5 can stimulate integrin dependent phosphorylation of cofilin, a change that favors dendritic actin polymerization [25].

Increases in the size of dendritic spines and associated increases in the number of glutamate receptors [26], [27], [28] can underlie increased responsiveness of the efferent neuron to transmitter quanta released presynaptically. Such responses, which can be measured as mini excitatory post synaptic currents (mEPSCs), will likely increase in both frequency and amplitude when spines enlarge, with frequency increases following from an increase in the number of responsive units and amplitude increases reflecting an increased number of AMPARs within a given unit. Synapses that are initially weak may be especially susceptible to change [29]. Changes in frequency and amplitude can in turn influence action potential probability. In support of this, a link between increased dendritic AMPARs and action potential probability has recently been described [30].

The multi-electrode array (MEA) records changes in electrical potential extracellularly, and more specifically action potentials from nearby units. It has been widely used to characterize dynamics from *in vitro* networks of neurons [31], [32], [33], [34], [35]. MEAs allow for simultaneous recordings to be obtained from many cells. Recordings can be acquired from cultures that are maintained for long periods of time, as well as cultures treated with stimuli that are difficult to administer *in vivo* or to slices.

In the present study we have used MEAs to record from hippocampal cultures with the goal of better understanding specific mechanisms by which MMPs influence neuronal excitability. At the same time, MEA recordings described herein have allowed us to examine the question of whether MMP activity can modulate important aspects of network activity and, in particular, burst dynamics.

## Materials and Methods

### Cell Culture

#### Ethics Statement

All experimental procedures were carried out in accordance with the Georgetown University Animal Care and Use Committee (GUACUC). Hippocampal tissue was harvested from embryonic day 18 Sprague-Dawley rats using a protocol modified from [36]. Briefly, neural tissue was finely chopped and digested with 0.1% trypsin and by mechanical trituration. Cells were plated onto multi-electrode arrays (MEA, Multi Channel Systems MCS GmbH, Reutlingen, Germany) that were previously treated with poly-d-lysine and laminin (Sigma, St. Louis, MO) at an approximate density of 600 cells/mm^2^. Cultures were maintained in Neuralbasal A medium with B27 (Invitrogen, Carlsbad, CA) with bi-weekly changes and stored in a humidified 5% CO_2_ and 95% O_2_ incubator at 37°C. Experiments were performed on cultures at 14 days *in vitro* (DIV).

### MEA recordings

Spontaneous electrical activity was recorded using a multi-electrode array (MEA). This MEA is composed of 59 titanium nitride electrodes, arranged on an 8×8 square array, and comprised of one reference electrode and four auxiliary analog channels each of which is 30 µm in diameter. The inter-electrode spacing is 200 µm. Upon plating, cells adhere to the silicon nitride substrate of the MEA and spontaneous electrical activity is detected after seven days. Electrical activity is amplified (MEA1060 preamplifier) and sampled at a 10 kHz acquisition rate in order to allow the detection of spikes. Data were digitized and stored on a Dell personal computer (Round Rock, TX) for offline analysis. Possible exposure to contaminants and fluctuations in osmolality and pH were significantly reduced during the data acquisition period by covering the MEA with a hydrophobic membrane that is permeable to CO_2_ and O_2_ [37]. Recordings were performed on a heated microscope stage at 37°C at 14 days *in vitro* (14DIV), because this is a time point during development where the network displayed vigorous spontaneous electrical activity and network connectivity is well-established [38]. To ensure reproducibility of results across animals, all reported experimental groups were derived from multiple experimental preparations. Results obtained from cultures within and across different preparations were not significantly different.

### Pharmacological Induction of LTP

We used the pharmacological agents forskolin (50 µM) and rolipram (100 nM) to induce chemical LTP. Forskolin was diluted in dimethyl sulfoxide (DMSO) to a stock concentration of 50 mM. Rolipram was dissolved in DMSO to a stock concentration of 100 µM. Both chemicals and DMSO were acquired from Sigma-Aldrich (St. Louis, MO).

On the day of the experiment, baseline electrical activity was recorded for 20 minutes. Next, to induce chemical LTP, 100 µL of conditioned media were removed from the MEA. 1 µL of each stock solution of forskolin and rolipram were added to the conditioned media and slowly added back into the MEA. 30 minutes following treatment, 6–10 minutes of continuous network activity was recorded for offline analysis.

To control for possible mechanical artifacts arising from the exchange of solutions, series of MEA recordings were performed on cultures in which conditioned media was removed and subsequently returned, but neither forskolin or rolipram were added. To control for solvent effects (DMSO), 1 µL of DMSO was diluted into the conditioned media prior to returning it to the MEA.

### Application of MMP inhibitors, anti-β_1_, and the soluble ICAM-5 ectodomain

The MMP inhibitors GM-6001 and (2R)-2-[(4-Biphenylylsulfonyl) amino]-3-phenylpropionic Acid (also known as MMP-2/9 inhibitor I or BiPS) (cat# 444241) were purchased from Calbiochem and used at a concentration of 2.5 µM. They were dissolved in DMSO to make a 2.5 mM stock solution. MMP inhibitors were added to cells 20 min. before control of cLTP recordings. Anti-β1 integrin was purchased from BD Pharmingen. This antibody was used as described [39] and has previously been shown to be neutralizing [39] and to inhibit soluble ICAM-5 stimulated phosphorylation of cofilin [25]. Recombinant ICAM-5 was purchased from R and D systems (Minneapolis, MN). This construct consists of leu31-arg828, followed by an IEGRMD linker and pro100-lys330 of human IgG1. An isotype matched immunoglobulin control (Santa Cruz Biotechnology) for the Ig portion of ICAM-5 was also used in select experiments. Cultures were treated with ICAM-5 or IgG control on an identical experimental timeline to cLTP, as described above.

### Western blot

Western blot was performed using hippocampal lysates as previously described [25]. Relative equivalency of protein loading and transfer across lanes was assessed by Ponceau staining prior to incubation of membranes with appropriate primary and secondary antibodies. Molecular weights were inferred by comparison to prestained markers (BioRad). Blots were probed with a commercially available polyclonal antibody to ICAM-5 (R & D Systems), which was raised against the major portion of the ectodomain (amino acids Leu 31 to Arg 828 of recombinant mouse ICAM-5), or to a C terminal, intracellular domain specific antibody produced in collaboration with Dr. Seung Lim [8].

### Data Analysis

All traces were high-pass filtered at 200 Hz to remove low frequency components. Next, extracellularly recorded spikes were detected using algorithm from Offline Sorter (Plexon Inc., Dallas TX), and thresholded at a multiple of the standard deviation (−5 s) of the biological noise. Due to significant changes in the shape of a spike during a burst resulting from changes in membrane excitability, no attempts were made to discriminate and sort spikes by electrode. In addition, this study concentrated on network activity that is suitability reflected in the overall, thresholded activity from each electrode without spike-sorting.

Network activity was then analyzed with proprietary software written in MATLAB (The MathWorks, Natick, MA). First, to investigate changes in overall network activity, we calculated the average firing rate, FR, over a binned (150 ms bin size), five-minute window for each electrode within the MEA.

Next, changes in a common temporal feature found in cultured networks, i.e. the burst, were investigated, as bursts can occur across the collective network. Following the spike detection process described above each electrode had a resulting spike train, τ_st_(t), expressed as:
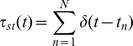
where N is the total number of spikes, 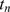 is the time of the *n*th spike and 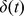 is a delta function that indicates a spike taking place at time 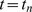. Next, the inter-spike interval (ISI) between spike *n* and spike *n*-1 (n>1) is:
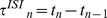



For all experimental groups, a “burst” from each electrode was defined to consist of no fewer than three spikes with a maximum inter-spike interval (ISI) of 150 ms. 150 ms was selected because it represented a time between a distribution of ISIs presumed to be within bursts and the intervals between bursts (see Baseline ISI distributions in Figures to follow). Lastly, the burst durations, Δ_i_, were defined as:
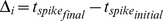



## Results

### MMP activity and β_1_ integrins contribute to cLTP evoked changes in spike counts detected by MEAs

MMP inhibitors have been shown to block LTP measured in rat hippocampal slices at area CA1 and evoked by stimulation of Schaffer collateral-commissural afferents through four trains of 100 Hz, 1 sec each and separated by 5 minutes [11]. MMP inhibitors also block cLTP associated changes in field EPSP slopes in rat hippocampal CA1 [11]. Chemical LTP paradigms stimulate changes, including spine enlargement, that are observed with tetanic stimulation protocols [40].

**Figure 1:**
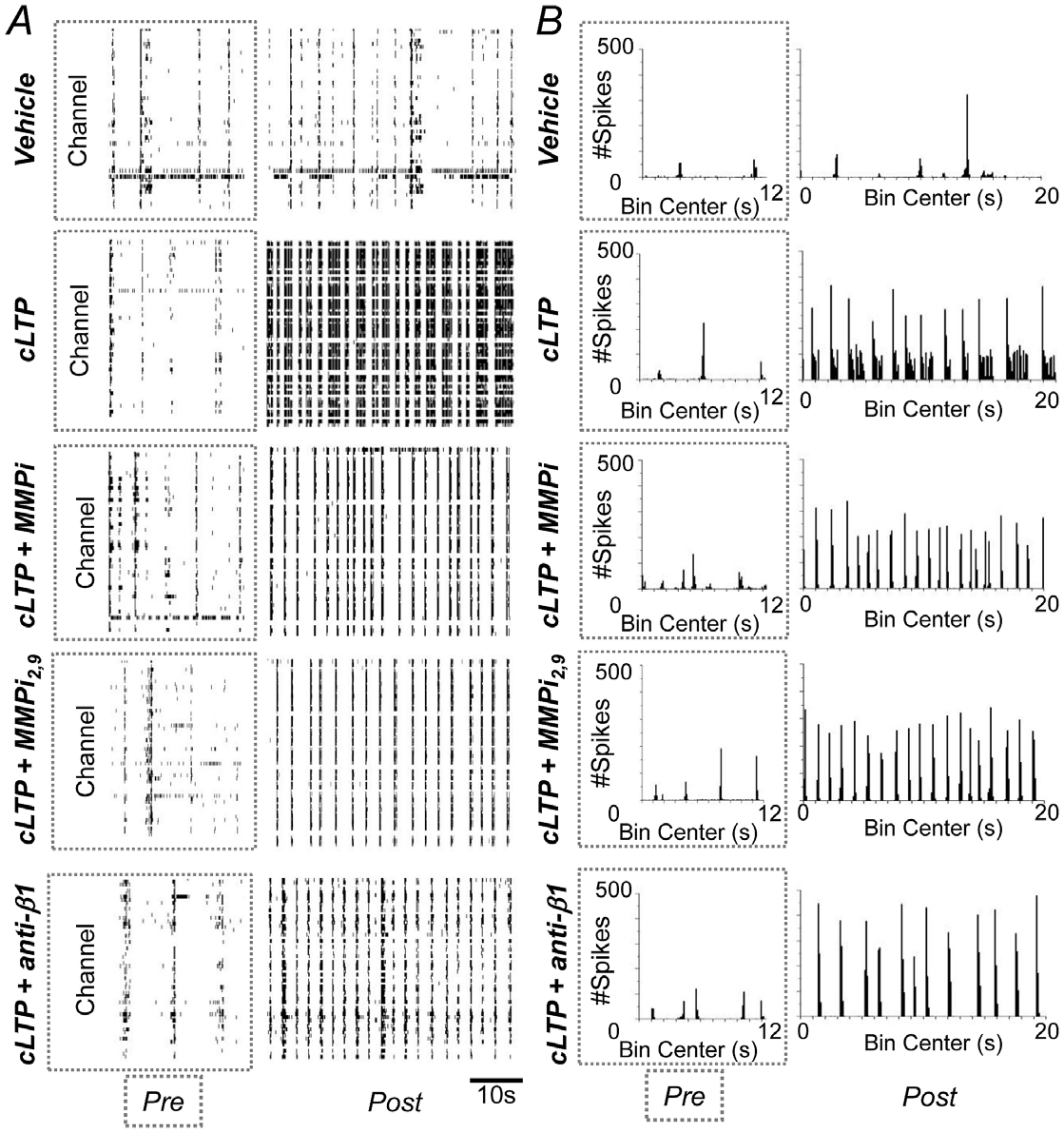
MMP activity and β_1_ integrins contribute to cLTP evoked changes in network firing rate. (A) Representative raster plots of network activity before (Pre) and following (Post) vehicle, cLTP, cLTP+GM-6001 (MMPi), cLTP+BiPS (MMPi_2,9_) or cLTP+anti-β_1_ treatment. The scale bar represents 10 seconds. (B) Total spike number (Y axis) as a function of binned (150 ms) intervals (X axis) for the four treatment groups. Differences in the average spike number per bin between the vehicle versus cLTP, as well as between cLTP and cLTP+MMPi or anti-β_1_ were significant (*p*<0.05, ANOVA, n = 4–6 per group).

To determine whether inhibition of MMP activity could reduce cLTP associated changes in action potential probability, we used MEAs to record from cultured hippocampal neurons. Two MMP inhibitors were tested, including a broad spectrum inhibitor (MMPi) and one that is more selective for MMP-2 and -9 (MMPi_2,9_). A neutralizing antibody to β_1_ integrins was also examined. Representative raster plots are shown in Figure 1A, while spike counts are shown in 1B. The average number of spikes per bin (unit time) in the cLTP paradigm was reduced by approximately 20% by the MMP inhibitors or the β_1_ integrin neutralizing antibody (vehicle: 6.18+/−0.45; cLTP: 51.8+/−1.2; cLTP+MMPi: 39.4+/−1.76; cLTP+MMPi_2,9_: 42.6+/1 2.9; cLTP+anti-β_1_: 38.1+/−1.4; MMPi alone: 5.83+/−0.82; MMPi_2,9_: 8.21+/−2.67; anti-β_1_ alone 5.29+/−1.36) The differences between average spikes per bin in the cLTP and cLTP with MMP inhibitors or anti-β1 integrin groups were statistically significant (p<0.01, ANOVA with Tukey's *post hoc* analysis). The difference between average spikes per bin in the vehicle and cLTP groups was also significant, as was the difference between vehicle and cLTP plus either MMP inhibitor or anti-β_1_, consistent with partial rather than complete inhibition (p<0.01). Differences between vehicle and inhibitors alone were not significant. Results are from 4–6 replicate experiments in vehicle control and cLTP+/−inhibitor groups, and from 3 replicate experiments in the inhibitor alone groups.

Spike count data (Figure 1B) shows that in the setting of cLTP, greatest spiking activity occurs after an initial period of relative quiescence. This is in line with work suggesting that action potential probability is influenced by an initial silent period as well as a sufficient level of excitation [41]. In addition, in the cLTP paradigm, the period in which spike counts are largest is followed by several shorter epochs of lesser but, in comparison to other groups, relatively increased spike activity. This activity may reflect a relatively increased level of excitability that exists following cLTP.

### MMP activity and β_1_ integrins contribute to cLTP associated changes in burst dynamics

**Figure 2:**
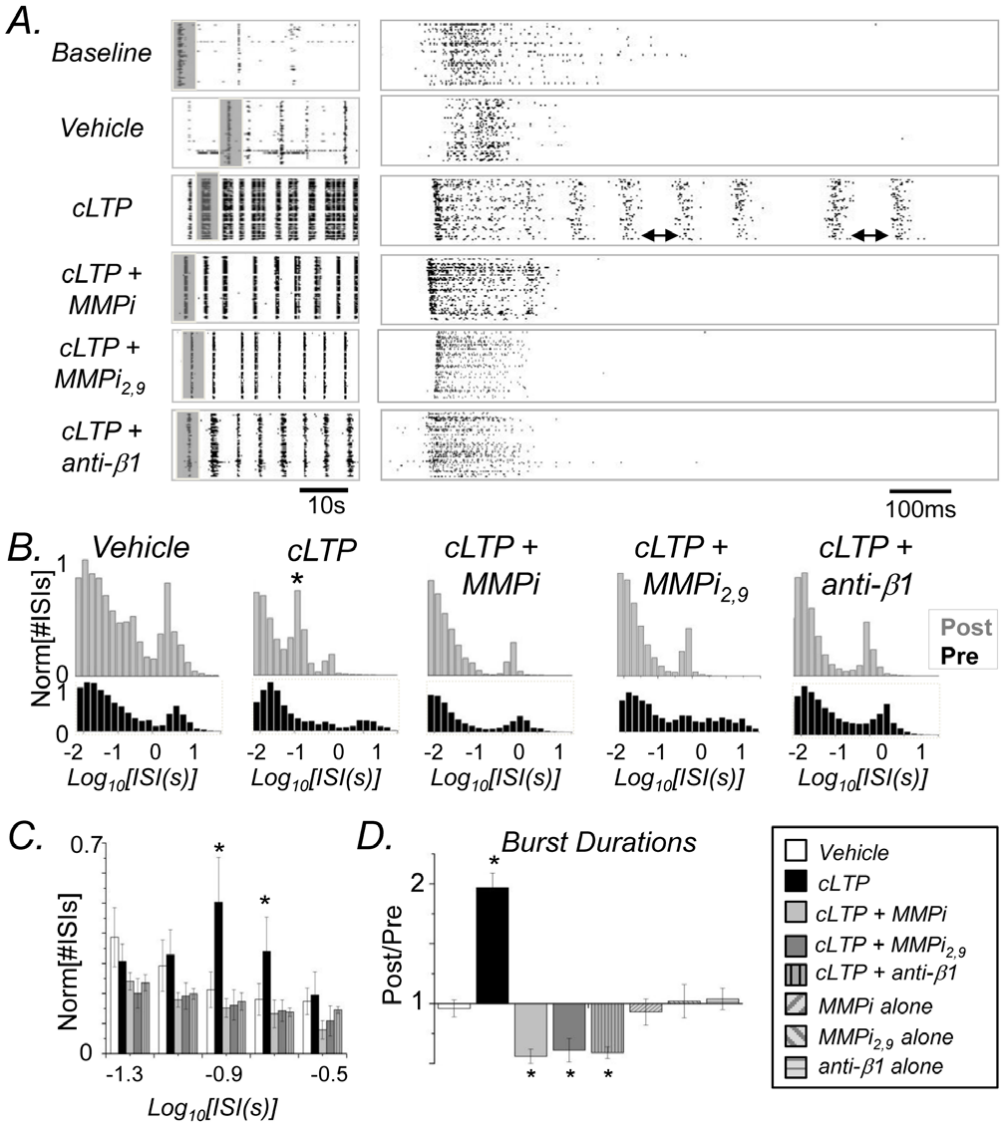
MMP activity and β_1_ integrins contribute to cLTP associated changes in interspike interval (ISI) distributions. (A) Raster plots showing expansions of representative “bursts” for each of the treatment groups as defined by a period of activity containing a maximum ISI of 150 ms (gray box in let panel is expanded in right panel). (B) Log histograms showing ISI distributions for each MEA represented in (A). ISIs within the left-most peaks (bin centers −2 to −1) likely represent ISIs within bursts while right-most peaks (bin center 0 to 1) represents ISIs for period between bursts. The peak at approximately -1 (cLTP, asterix) likely represents the ISI between burstlets within a superburst (illustrated by arrows in 2A). Data were normalized to the bin containing the largest number of ISIs for each culture. (C) Expansions of log histograms show 2 time points, denoted by an asterix, for which cLTP shows a statistically significant increase in normalized ISI values as compared to control or cLTP with inhibitors (*p*<0.05, ANOVA). (D) Burst duration is also significantly increased by cLTP treatment in an MMP or β_1_ integrin dependent manner (*p<0.001 compared to control, ANOVA; data are presented as the mean and SEM of the ratios of pre to post treatment values and represent results from 4–6 replicates for vehicle and cLTP groups and 3 replicates from inhibitor alone groups).

Rhythmic bursting activity is important to information propagation and memory consolidation [41]. To determine whether inhibition of MMP activity or β_1_ integrin signaling can alter cLTP dependent effects on burst dynamics, bursts were examined as a function of treatment group. While a precise burst definition can vary, and is influenced by culture preparation and experimental conditions, bursts are generally defined as brief periods during which the spike rate detected in many electrodes is several fold greater than baseline [38]. Representative raster plot data with activity shown in an approximate 10 sec interval, and also in an expanded 100 msec interval, is shown in Figure 2A. Each tick mark corresponds to an action potential and bursts can be observed as patterns of high frequency spiking activity.

Representative raster plots show that cLTP is associated with “superbursts” in which a large number of small bursts occur within tight clusters (inter-cluster intervals are typically 10× longer than intracluster intervals) [38]. A representative superburst can be observed in the expanded window for the cLTP treatment (Figure 2A, third column). Superbursts occur with strong stimulus efficacy [42], and may thus be favored by spine enlargement. Superbursts might also be favored when synapse number increases [43], as might occur with cLTP stimulated input from previously silent synapses [44].

Superbursts were observed only in the cLTP treatment group. Superbursts were not observed following pretreatment of cultures with MMP inhibitors or a neutralizing antibody to β_1_ integrins. Consistent with this, a log histogram of interspike intervals (Figure 2B) shows a prominent ISI peak at approximately 100 msec (asterisk) that appeared only in the cLTP group, and represents the ISI between spikes at the end one and beginning of another spike cluster, or burstlet, occurring within the superbursts (Figure 2A, arrows). In the expanded log ISI histogram shown in 2C, in which normalized ISIs having a given time value are presented for the four treatment groups, the difference between cLTP and other groups was significant at the two time points indicated by an asterisk (*p*<0.05, ANOVA).

Finally, the emergence of superbursts in the cLTP-treated cultures represents a significant increase in the epoch of increased action potential occurrence, or duration of the burst, over vehicle treatment as shown in Figure 2D (p<0.001; ANOVA Tukey's post hoc comparison). Alternatively, cLTP following pretreatment of cultures with MMP inhibitors or the β_1_ neutralizing antibody will significantly shorten burst durations compared to vehicle treatment (Figure 2D; p<0.001; ANOVA Tukey's post hoc comparison). On their own, the MMP inhibitors and anti-β_1_ were without significant effect on burst duration (Figure 2D).

### The MMP generated integrin binding ligand, soluble ICAM-5, stimulates a dose dependent increase in spike counts

**Figure 3:**
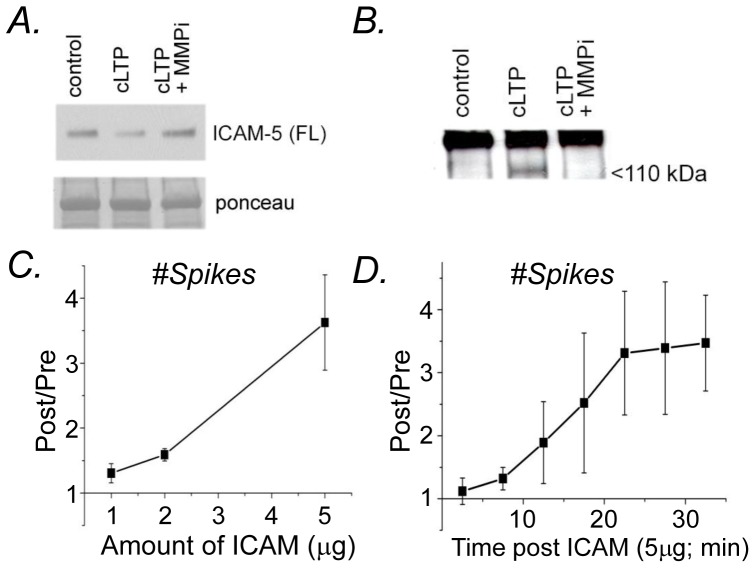
Soluble ICAM-5 is generated with cLTP and stimulates a dose dependent increase in spike counts. A) and B) Western blot analysis of lysates from control, cLTP, and cLTP+MMPi treated hippocampal neurons. (A) cLTP stimulates a reduction in immunoreactivity for full length (FL) ICAM-5, as detected by an antibody to the ectodomain and inferred from apparent molecular weight (∼140 kDa), that is abrogated by MMP inhibition (MMPi). Ponceau staining, showing equal transfer of total protein at approximately 50 kDa, is shown below. (B) A separate set of control and treated lysates, tested by Western blot and probed with a relatively sensitive C terminal specific antibody [8], shows a previously described 110 kDa ICAM-5 cleavage product [25] in the cLTP lane (arrowhead). (C) Treatment with increasing amounts of soluble ICAM-5 result in a relative increase in spike number. Differences between the 1 and the 5 µg/ml doses were significant (n = 4 per group, *p*<0.05, ANOVA; data are presented as the mean and SEM of the ratios of pre to post treatment values). (D) Spike number as a function of time for the 5 µg dose.

In previous work, we have demonstrated that the ectodomain of ICAM-5 is shed in a neuronal activity dependent manner [8]. NMDA stimulation of cultured hippocampal neurons, and high frequency stimulation of hippocampal slices, stimulate MMP dependent shedding of this molecule [8]. Cleavage at two sites in the ectodomain has been demonstrated, leading to generation of the near full length ectodomain as well as two smaller fragments which both contain Ig-like domains [19], [20]. Shown in Figure 3A and B are Western blot results from hippocampal culture lysates prepared at the conclusion of MEA recordings (40–45 minutes post treatment). A cLTP associated reduction in full length ICAM-5 can be appreciated (Figure 3A) and a previously described [25] MMP generated cleavage fragment can be observed at approximately 110 kDa (Figure 3B).

In previous work we have also shown that the ICAM-5 ectodomain can interact with integrins known to be expressed on dendritic spines, and that this ectodomain can stimulate phosphorylation of cofilin [25], a biochemical change that allows for actin polymerization. Herein we have tested the potential for the ICAM-5 ectodomain to increase neuronal excitability as detected by the MEA system. As shown in Figure 3C, the ectodomain stimulated a dose dependent increase in firing rate. The difference between the 1 µg/ml and 5 µg/ml dose was statistically significant at *p*<0.05 (ANOVA with Tukey's *post-hoc* comparison). In Figure 3D we show spike number as a function of time for the 5 µg/ml dose.

### Soluble ICAM-5 stimulatesβ_1_ integrin dependent changes in network and burst activity

**Figure 4:**
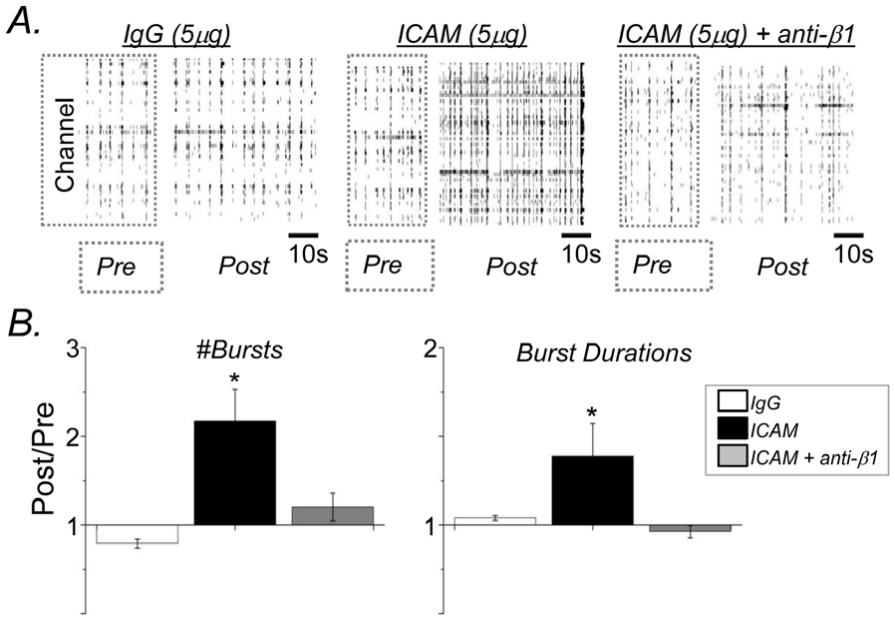
ICAM-5 stimulatesβ_1_ integrin dependent changes in network and burst activity. (A) Representative raster plot sowing spike data for control (IgG), ICAM-5 and ICAM-5+anti-β_1_. (B) Burst number and duration (mean and S.E.M.) are shown. The difference between burst number in control and ICAM-5 groups, as well as the difference between burst duration in ICAM-5 and ICAM-5+anti-β_1_ was significant (p<0.05, ANOVA, n = 4 per group).

We also examined burst activity as affected by the ICAM-5 ectodomain. In addition, we tested the neutralizing antibody to β_1_ on ICAM-5 stimulated effects on network and burst activity. Results are shown in Figure 4 and suggest that ICAM-5 associated changes in network excitability are abrogated by the neutralizing antibody, as observed in representative raster plots (Figure 4A) and in the number of bursts and their durations (Figure 4B). By ANOVA, the difference between burst number in control and ICAM-5 treated cultures, as well as the difference between burst duration in sICAM-5 versus ICAM-5+anti-β1 treated cultures, was significant at *p*<0.05.

Of interest is that while we observed a trend towards increased burst duration with ICAM-5, we did not observe the occurrence of superbursts in association with this stimulus. It is possible that cLTP provides a stronger stimulus for spine expansion and/or that cLTP stimulates additional effects, independent of those mediated by ICAM-5 or achieved by spine expansion, to increase neuronal excitability.

## Discussion

Emerging evidence suggests that MMPs play a role in hippocampal dependent learning and memory, addiction, and LTP [11], [13], [45], [46]. While the mechanisms by which MMPs influence learning and memory are not fully understood, one possibility is that these enzymes cleave synaptic adhesion molecules to release N terminal fragments that go on to interact with previously unengaged integrins. Synaptic CAMs are ideally positioned to be targeted by MMPs that are released and/or activated in a neuronal activity dependent manner. Consistent with this possibility, we have shown that the N terminal fragment of at least one CAM can stimulate β_1_ dependent phosphorylation of cofilin, an event that would favor an increase in the size of dendritic spines [25]. An increase in spine size has in fact been observed following treatment of hippocampal neurons with exogenous MMP-9 [12].

An increase in spine size has been linked to an increased number of post-synaptic AMPA receptors [26]. This change may in turn increase the amplitude and/or frequency of excitatory post synaptic potentials (EPSPs) [30]. An increase in frequency would be expected with AMPAR incorporation into synapses which were previously silent due to a relative absence of AMPARs [28], [44]. An increase in EPSP amplitude and/or frequency would in turn be expected to increase the probability of action potential occurrence and thus spiking activity detected by the MEA system. And while the regulation of burst probability and duration is complex, with the two often showing an inverse correlation [47], increases in action potential probability will generally lead to an increase in burst occurence [41], [47], [48].

In the current study, we examined cLTP evoked changes in spiking activity and burst dynamics as affected by inhibitors of MMPs or β_1_ integrin signaling. We also examined a potential mechanism by which MMPs and β_1_ integrins can influence network dynamics. In particular, we examined the ectodomain of ICAM-5, a product of MMP proteolysis, for its ability to stimulate integrin dependent changes in network activity parameters. Inhibitors reduced cLTP stimulated spike activity. That this reduction was not complete is consistent with the possibility that the cLTP paradigm increased these endpoints by varied mechanisms including one or more that are MMP and β_1_ independent. Experiments with the ICAM-5 ectodomain also supported the hypothesis that MMPs and β_1_ integrins can increase neuronal excitability. This product of MMP proteolysis significantly increased spike and burst number. Moreover, blocking β_1_ integrins with neutralizing antibodies diminished ICAM-5 dependent effects on these parameters.

We also observed that inhibition of MMP activity or β_1_ integrin signaling had a substantial effect on cLTP associated changes in burst dynamics. Of particular interest is that both the MMP inhibitor and neutralizing antibody to β_1_ integrins abolished cLTP associated superbursts, and an associated intraburst rhythmic activity in the theta range. Since stimulated theta activity can be used to induce synaptic potentiation [49], [50], [51], changes to which chemically evoked MMP activity contribute might be part of a positive feedback mechanism in which widespread but synaptically localized changes favor the emergence of rhythmic activity that further facilitates synaptic potentiation. ICAM-5 also stimulated an increase in firing rate and burst number, suggesting that MMP generated CAM fragments might contribute to MMP dependent effects on neuronal excitability. Although soluble ICAM-5 treatment was not associated with the emergence of superbursts characteristic of cLTP, a phenomenon that may require a stronger stimulus for spine expansion and/or effects independent from those stimulated by ICAM-5, this CAM fragment did increase burst duration suggesting that it too could increase the duration of relatively increased excitability.

The potential for MMPs to influence not only spike but burst dynamics is significant. The downstream effects of a burst are considered to be stronger than those of a single spike [41]. While we examined bursting at the single electrode detection level, from Figure 2 it is apparent that bursting increased across the population of cells. Bursting on a single cell and/or population level may be important to varied processes including the development of networks, pulsatile hormone release, facilitated information propagation, and memory consolidation [52], [53], [54], [55]. With respect to the latter, sedatives or anaesthetic agents administered in amounts insufficient to promote unconsciousness, but sufficient to disrupt rhythmic brain activity, have been associated with amnesia [56]. In addition, slow wave sleep, which is characterized by a particular rhythmic activity, has been linked to reactivation of neuronal activity patterns from preceding behavior in rat cortex and hippocampus [57]. Bursting activity may strengthen synapses that have previously been strengthened through other means.

As compared to stimuli that may target spines and other effectors of neuronal excitability in a more restricted or circuit specific manner, cLTP stimulation of cultured hippocampal neurons would be expected to have relatively diffuse or widespread effects. This stimulus may nonetheless have *in vivo* relevance. For example, when modestly elevated, norepinephrine, which like cLTP can increase adenylate cyclase activity, can enhance memory. Levels of this neurotransmitter may be diffusely altered with anxiety or in association with specific stages of the sleep/wake cycle. Norepinephrine is concentrated in neurons of the locus coeruleus which project in a widespread manner to hippocampus and cortex [58], [59], [60]. Though highly speculative, it is possible that norepinephrine could increase rhythmic bursting to strengthen synapses that were previously strengthened through the learning of a specific task.

While previous studies have implicated localized and regulated proteolysis in the learning of specific tasks, such as finding the Morris water maze platform [61], data herein suggest that these enzymes may also play a role in facilitating rhythmic changes that are evoked by a widely applied/received stimulus, of which the cLTP cocktail is an example. An outstanding question relates to the extent to which emergent widespread bursting can be engendered by the same stimuli that are important to formation of smaller units such as synapses or discrete circuits that widespread bursting serves to enhance. Varied molecular mechanisms may contribute to both increased efficacy of neurotransmission at specific synapses as well as to widespread rhythmic activity. For example, pyramidal cell dendritic calcium spikes contribute to one form of *in vitro* theta activity [62]. For a relatively complete discussion of factors that influence rhythmic activity, including factors relevant to pyramidal cells and chemical synapses in particular, please see [63].

Understanding the mechanisms by which MMPs, and MMP generated integrin binding ligands in particular, contribute to changes in burst dynamics will require further study. Nonetheless, effects observed herein are consistent with those expected to result from dendritic spine maturation. Additional support for the hypothesis that spine changes play a role is published data showing that EphA4 expression can promote both spine maturation and increased burst duration [64]. It should be noted, however, that like cLTP, MMPs may have relevant effects that are independent of spine size and/or AMPARs. For example, integrin binding ligands may increase NMDAR phosphorylation and function through a src kinase dependent mechanism [65].

In terms of specific MMP generated integrin binding ligands, it is unlikely that ICAM-5 is the only CAM that is shed in a neuronal activity dependent manner to stimulate spine expansion. Though not yet tested for effects on neurotransmission, additional CAM ectodomains that are released with neuronal activity include nectin-1, L1 and NCAM [66], [67], [68], [69], [70], [71]. Of interest is that several CAM ectodomains, including that of L1, can interact with integrins [70], [72].

In conclusion, our data suggest that MMPs and soluble ICAM-5 have the potential to influence neuronal excitability and burst dynamics, with both stimulating an overall increase in excitability of the system as determined by an increase in overall spike counts, burst number, and/or burst duration. Future experiments with enhanced synaptic release of MMPs as potentially mediated by viral vector delivery, or experiments with MMP inhibitors prior to differing stimuli for the induction of LTP, are warranted. In addition, future studies with CAM cleavage resistant mutants will be necessary to determine the extent to which cleavage of specific CAMs *per se* plays a role. Given that varied MMP and CAM polymorphisms have been linked to altered learning and memory or propensity for addiction [73], [74], [75], future experiments that examine network dynamics in cultures expressing these polymorphisms may also be of interest.
